# Polar Invasion and Translocation of *Neisseria meningitidis* and *Streptococcus suis* in a Novel Human Model of the Blood-Cerebrospinal Fluid Barrier

**DOI:** 10.1371/journal.pone.0030069

**Published:** 2012-01-11

**Authors:** Christian Schwerk, Thalia Papandreou, Daniel Schuhmann, Laura Nickol, Julia Borkowski, Ulrike Steinmann, Natascha Quednau, Carolin Stump, Christel Weiss, Jürgen Berger, Hartwig Wolburg, Heike Claus, Ulrich Vogel, Hiroshi Ishikawa, Tobias Tenenbaum, Horst Schroten

**Affiliations:** 1 Pediatric Infectious Diseases, Department of Pediatrics, Medical Faculty Mannheim, Heidelberg University, Mannheim, Germany; 2 Department of Statistics, Medical Faculty Mannheim, Heidelberg University, Mannheim, Germany; 3 Max Planck Institute of Developmental Biology, Tübingen, Germany; 4 Institute of Pathology, University of Tübingen, Tübingen, Germany; 5 Institute for Hygiene and Microbiology, University of Würzburg, Würzburg, Germany; 6 Department of Anatomy, Jikei University School of Medicine, Tokyo, Japan; Health Protection Agency, United Kingdom

## Abstract

Acute bacterial meningitis is a life-threatening disease in humans. Discussed as entry sites for pathogens into the brain are the blood-brain and the blood-cerebrospinal fluid barrier (BCSFB). Although human brain microvascular endothelial cells (HBMEC) constitute a well established human *in vitro* model for the blood-brain barrier, until now no reliable human system presenting the BCSFB has been developed. Here, we describe for the first time a functional human BCSFB model based on human choroid plexus papilloma cells (HIBCPP), which display typical hallmarks of a BCSFB as the expression of junctional proteins and formation of tight junctions, a high electrical resistance and minimal levels of macromolecular flux when grown on transwell filters. Importantly, when challenged with the zoonotic pathogen *Streptococcus suis* or the human pathogenic bacterium *Neisseria meningitidis* the HIBCPP show polar bacterial invasion only from the physiologically relevant basolateral side. Meningococcal invasion is attenuated by the presence of a capsule and translocated *N. meningitidis* form microcolonies on the apical side of HIBCPP opposite of sites of entry. As a functionally relevant human model of the BCSFB the HIBCPP offer a wide range of options for analysis of disease-related mechanisms at the choroid plexus epithelium, especially involving human pathogens.

## Introduction

Meningitis is a life-threatening disease in humans leading to severe illness and death world-wide. A major cause of acute meningitis is the infection with encapsulated bacteria [1], [2]. To induce inflammation within the brain, the pathogens need to cross the blood-brain barriers, which include the “classical” blood-brain barrier (BBB) represented by the endothelial cells within central nervous system (CNS) microvessels as well as the blood-cerebrospinal fluid (CSF) barrier (BCSFB) that is formed by the epithelial cells of the choroid plexus [3]. Required for barrier formation are dense connections between cells, which are set up by continuous strands of tight junctions (TJs). TJs are molecular structures consisting of transmembrane proteins including the claudins and occludin as well as additional intracellularly associated membrane proteins like zonula occludens (ZO)-1, which connect the TJs to the actin cytoskeleton [4], [5]. Further connections between the cells are mediated by the less tight adherence junctions (AJs) involving the transmembrane protein E-cadherin [6].

The barrier-building cells display certain characteristics, which enable them to restrict exchange across the cell layers to a minimum. In case of the endothelium presenting the BBB these cells are interconnected by a dense network of TJs and they exhibit a low pinocytotic activity concomitant with the absence of fenestrae [7]. In the highly perfused choroid plexus the endothelial cells of the blood vessels are fenestrated and without TJs; instead an unique system of TJs between the cells of an outer epithelial layer provides the morphological correlate of the BCSFB [8]. Properties of these cellular barriers are a high transendothelial or transepithelial electrical resistance (TEER) as well as a low permeability for macromolecules [9]. During inflammatory events these barriers undergo major alterations, which lead to opening of TJs, break-down of barrier function and massive influx of immune system cells into the brain [3], [8].

Despite the significant morbidity and mortality of bacterial meningitis the pathogenesis of meningitis in humans is still incompletely understood. An important factor for investigating this disease is the development of suitable *in vitro* systems mimicking the abovementioned barriers. Whereas human models of the BBB employing immortalized cell lines have been developed [10]–[12], *in vitro* systems mimicking the BCSFB are limited to animal models, including rat cell lines and primary porcine choroid plexus epithelial cells (PCPEC) [13]–[17] (an extensive recent review covering BCSFB in vitro models is provided by Strazielle and Ghersi-Egea [18]). A described cell line derived from human choroid plexus carcinoma [19], [20] lacks crucial properties of a reliable BCSFB model as the formation of continuous TJs [21]. Therefore, an urgent need for the development of a human BCSFB model system exists. Recently a human choroid plexus papilloma cell line (HIBCPP) was established [22], but has not yet been characterized for its suitability as BCSFB model system.

Organisms causing bacterial meningitis in mammals, for which a role of the BCSFB has been described, include *Neisseria meningitidis* (*N. meningitidis*), *Escherichia coli* (*E. coli*) and the zoonotic pathogen *Streptococcus suis* (*S. suis*) [1], [23], [24]. Lesions at the choroid plexus have been observed in naturally and experimentally induced cases of meningitis caused by *S. suis* in pigs and mice [24]–[27]. Employing primary porcine choroid plexus epithelial cells (PCPEC) as model system, we have recently demonstrated polar invasion and translocation of *S. suis* across the BCSFB from the basolateral side. During this process the presence of the bacterial capsule, a described virulence factor of *S. suis*, played an important role [28]. Although recent outbreaks of meningitis caused by *S. suis* infection have been described in Southeast Asia, *S. suis* is not very relevant in western countries [29], [30]. Importantly, evidence for association with the choroid plexus has also been shown for *N. meningitidis* and *E. coli* in humans [31], [32], further supporting the need for a human cell-based *in vitro* model of the BCSFB.

In the present study, we have investigated the suitability of HIBCPP as a model system of the BCSFB. We demonstrate that HIBCPP, when cultured under appropriate conditions, display several features of a functional BCSFB including the formation of TJs and the development of a high TEER concomitant with a low permeability for macromolecules when they are grown on transwell filters. Using HIBCPP as a model system for the BCSFB we show that the zoonotic pathogen *S. suis* as well as the human specific bacterium *N. meningitidis* invade HIBCPP in a polar fashion from the basolateral side. Entry of *N. meningitidis* into HIBCPP is strongly attenuated by the presence of a capsule and transmigrated meningococci form microcolonies at the apical side of HIBCPP.

## Results

### HIBCPP develop a barrier function on transwell filters *in vitro*

**Figure 1:**
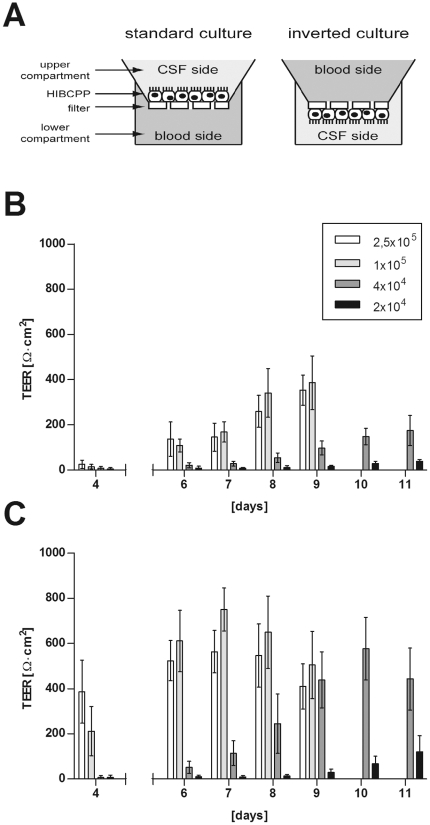
HIBCPP develop high TEER in standard and inverted transwell filter systems. Throughout this study cells were grown either on the upper side (standard transwell filter system) or the lower side (inverted transwell filter system) of the filter supports (**A**; schematic representation). For experiments (**B**, **C**) HIBCPP were seeded on transwell filters in the amounts indicated in the legend. Cells were cultivated either in the standard transwell filter system (**B**) or the inverted transwell filter system (**C**). TEER was measured over time at the days after seeding of the cells as indicated on the x-axis. Shown is the mean+/−SD of four (standard culture) or five (inverted culture) experiments, respectively, each performed in triplicates.

We have previously used PCPEC to examine bacterial meningitis and inflammatory events at the BCSFB *in vitro* [28], [33], [34]. During these studies we developed an “inverted” transwell filter system to investigate bacterial invasion and translocation from the physiologically relevant basolateral (blood) to the apical (CSF) side (Fig. 1 A). In these experiments the PCPEC exhibited characteristics of a functional BCSFB as the formation of a transepithelial membrane potential, which can be determined by the development of a high TEER [28].

A human choroid plexus papilloma cell line (HIBCPP) has been described recently. HIBCPP are characterized as an epithelial arrangement and are positive for keratin staining [22]. To investigate the applicability of HIBCPP as human model system for the BCSFB we grew HIBCPP on transwell filters employing the standard system as well as the inverted transwell filter system and determined the TEER values over time. As can be seen in Fig. 1A and Fig. 1B the HIBCPP developed a high membrane potential under both culture conditions. The time-point a detectable TEER started to develop depended in both cases on the amount of cells seeded at the beginning of experiment. TEER values reached up to about 500Ω×cm^2^ in the standard transwell filter system (Fig. 1B) and up to about 800Ω×cm^2^ in the inverted transwell filter system (Fig. 1C). About 3 days after cells reached a high TEER a decline of TEER values could be observed (2.5×10^5^ and 1×10^5^ cells seeded in Fig. 1C and data not shown).

**Figure 2:**
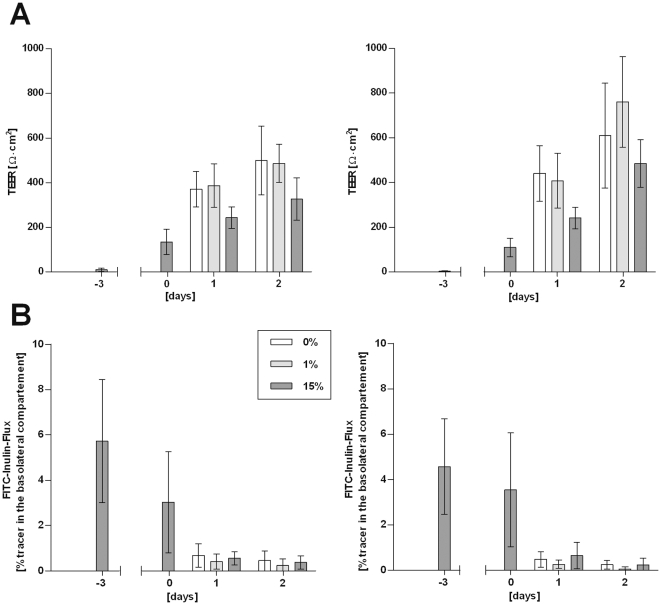
High TEER values correlate with low FITC-inulin flux through HIBCPP-layers. HIBCPP were grown until a TEER above 70Ω×cm^2^ was measured (day 0) and subsequently cultured in 15%, 1%, or 0% FCS, respectively, as indicated. At the indicated days TEER (**A**) and the FITC-inulin flux (**B**) were determined. Cells were grown in the standard transwell filter system (1×10^5^ cells; left panels) or the inverted transwell filter system (4×10^4^ cells; right panels). Shown is the mean+/−SD of eight experiments performed in triplicates.

It has been described for the PCPEC-based model system that serum withdrawal after confluency leads to enhanced barrier properties including higher TEER values [14]. To elucidate whether a similar effect can be observed with HIBCPP we grew the cells in the standard as well as the inverted transwell filter system until a TEER above 70Ω×cm^2^ was measured (designated day 0 in Fig. 2) and subsequently continued to cultivate the cells in medium containing either 15%, 1% or 0% FCS, respectively. Both in the standard and inverted transwell filter system HIBCPP reached higher TEER values when cell culture was continued in 1% or 0% FCS following day 0 compared to continued growth in 15% FCS (Fig. 2A). Differences were statistically significant for both cells grown in 1% FCS or 0% FCS compared to 15% FCS on day 1 and day 2 in the standard culture system (day 1: each p<0.0001; day 2: p = 0.0010 for 1% FCS and p = 0.0016 for 0% FCS). In the inverted culture system, differences were statistically significant for both growth in 1% FCS and 0% FCS compared to 15% FCS on day 1 (each p<0.0001), but only for growth in 1% FCS on day 2 (1% FCS: p<0.0001, 0% FCS: p = 0.1158; Fig. 2A).

A typical hallmark of the functional BCSFB is a low permeability for macromolecules [14], [15]. We therefore investigated whether HIBCPP layers grown on transwell filters developed an impermeability for FITC-labelled inulin (FITC-inulin; a small sugar with an average molecular weight of 3000–6000) concomitantly with the formation of a high TEER. As demonstrated in Fig. 2B HIBCPP layers allow a high FITC-inulin flux (3% and higher) up to day 0 when TEER values are still low. Subsequently and simultaneously with the development of a high TEER, permeability for FITC-inulin drops to levels below 1%. This decrease in macromolecular permeability can be observed with all three serum concentrations (15%, 1%, 0% FCS) and can be detected in the standard as well as the inverted transwell filter system.

### HIBCPP express junction proteins and develop tight junctions

**Figure 3:**
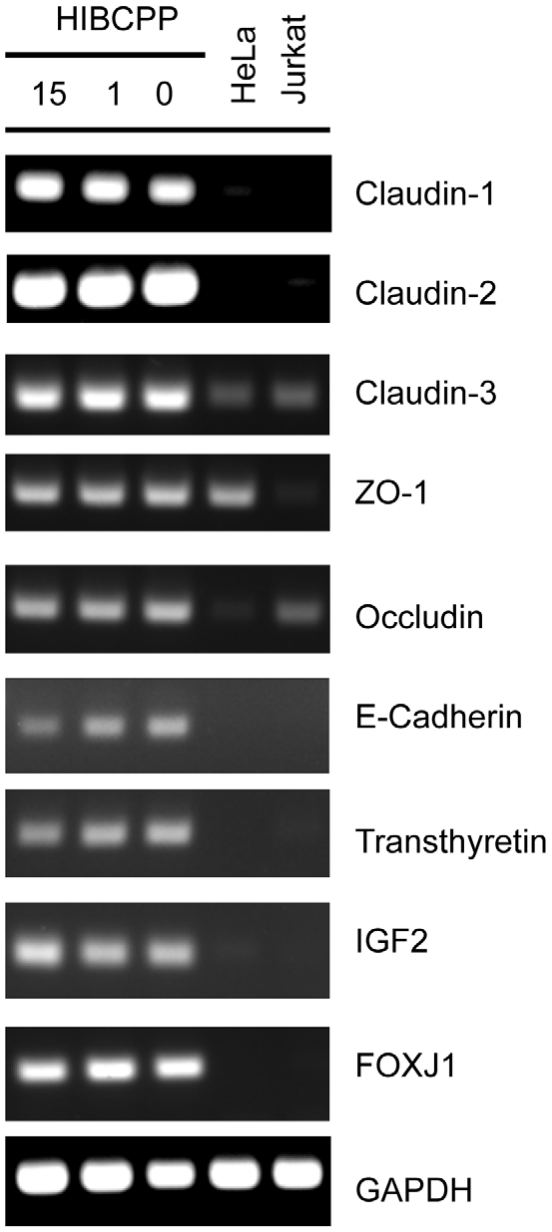
RT-PCR analysis of the expression of the genes encoding junctional proteins and of the choroid plexus markers Transthyretin, IGF2 and FOXJ1 in HIBCPP. HIBCPP were grown in 6well plates until confluency and subsequently cultured for 1 day in medium containing 15, 1 or 0% FCS as indicated at the top of the lanes. The expression of the genes indicated at the right was analysed by RT-PCR. For comparison, RNA isolated from HeLa and Jurkat cells was analysed as well. Expression of the GAPDH gene served as control. The results shown are a typical example from three independently performed experiments.

The polarization of epithelial cells and the regulation of their barrier function is achieved by the expression of AJ and TJ proteins [7], [35]. To investigate if AJ and TJ components are present in HIBCPP we determined expression of a typical AJ protein (E-cadherin) and of several TJ associated factors (Claudin-1, -2, -3, ZO-1, Occludin) by RT-PCR. As can be seen in Fig. 3 all the corresponding genes were expressed in HIBCPP as assessed by RT-PCR. Also Transthyretin, insulin-like growth factor 2 (IGF2) and forkhead box J1 (FOXJ1), which are marker proteins for choroid plexus epithelial cells [36]–[39], were detected. We found qualitatively similar expression levels of all factors analysed when RNA isolated from HIBCPP cultured under different serum conditions (15%, 1% or 0% FCS after confluency) was examined (Fig. 3).

**Figure 4:**
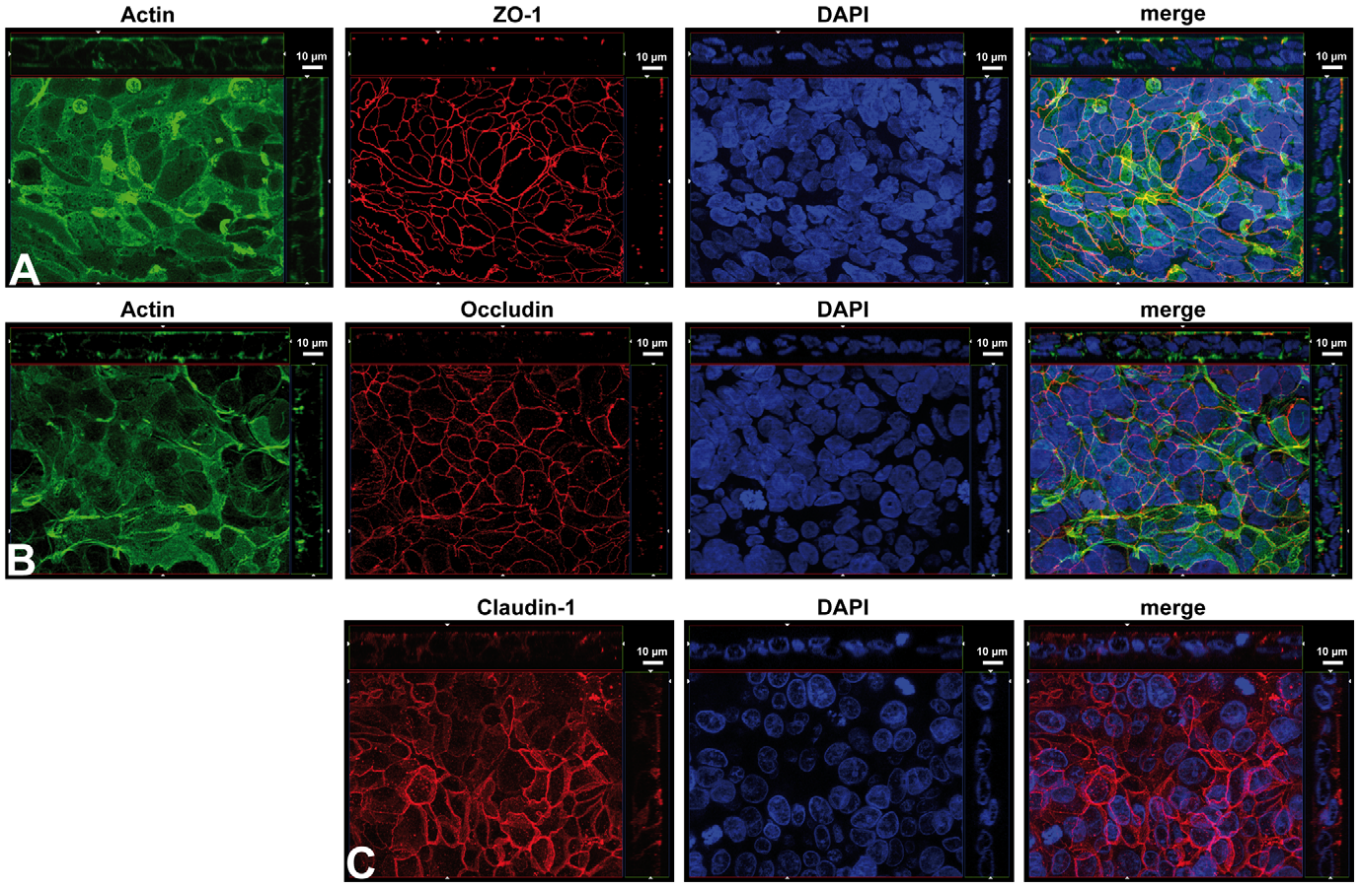
HIBCPP display continuous tight junction strands. HIBCPP grown in the inverted transwell filter system were stained for detection of ZO-1 (**A**), Occludin (**B**) and Claudin-1 (**C**). Pictures presented are Apotome-generated images; *bottom* of each panel is an *xy* en face view of a cell culture monolayer shown in a maximum-intensity projection through the z-axis; *top and side* of each panel is a cross section through the z-plane of multiple optical slices. The apical side of HIBCPP is oriented towards the top or the right side, respectively, of the top and side images of each panel. In A and B the actin cytoskeleton was in parallel stained with phalloidin-FITC. Since Claudin-1 samples were fixed with methanol we could not observe a qualitatively sufficient actin staining. In all samples nuclei were stained with DAPI. The images shown are representative example of multiple stainings.

To collect information concerning the tight junction morphology of HIBCPP we analyzed HIBCPP layers grown in the inverted (Fig. 4) and standard (data not shown) transwell filter system by immunofluorescence against ZO-1 (Fig. 4A), Occludin (Fig. 4B) and Claudin-1 (Fig. 4C). Cells were grown until TEER values were above 70Ω×cm^2^ and subsequently cultivated for one more day in medium containing 1% FCS. Cells prepared for immunofluorescence displayed a TEER around 500Ω×cm^2^. Corresponding to the RT-PCR data, all three investigated proteins were expressed and detectable on protein level (Fig. 4). Importantly, the immunofluorescence-produced signal of the three TJ-associated factors displayed a continuous pattern localized at the sites of cell-cell contact. This staining is largely located at the apical region of the cell-cell contacts, although in the case of Occludin and of Claudin-1 some signal at more basolateral areas of the cell-cell borders could also be detected (Fig. 4B,C). Cells grown in 15% FCS or 0% FCS showed similar results suggesting that the presence of serum factors is dispensable for protein expression (data not shown).

The *xy* en face view of the Apoptome images (Fig. 4) revealed a “puzzle-like” structure of the HIBCPP cell layer. Staining of the nuclei and of the actin-cytoskeleton showed that the cells tend to overlap each other (pile up), as has been noted in the original description of the HIBCPP [22]. Generally the HIBCPP display a dense distribution with rather small amounts of cytoplasm around the nucleus, although cells sometimes possess large cytoplasmatic extensions (Fig. 4). In most regions of the filters only a single continuous layer of tight junctions could be detected indicating formation of a functional monolayer. In some areas fragments of a second layer of TJ strands were noticed below the continuous upper layer (Fig. 4). This observation matches the mentioned ability of the cells to grow in multilayers and to form papillary-like structures [22]. The fragmentary appearance of the second layer of TJ strands in only small areas of the filter membranes is in agreement with the majority of the HIBCPP being present as a functional monolayer.

**Figure 5:**
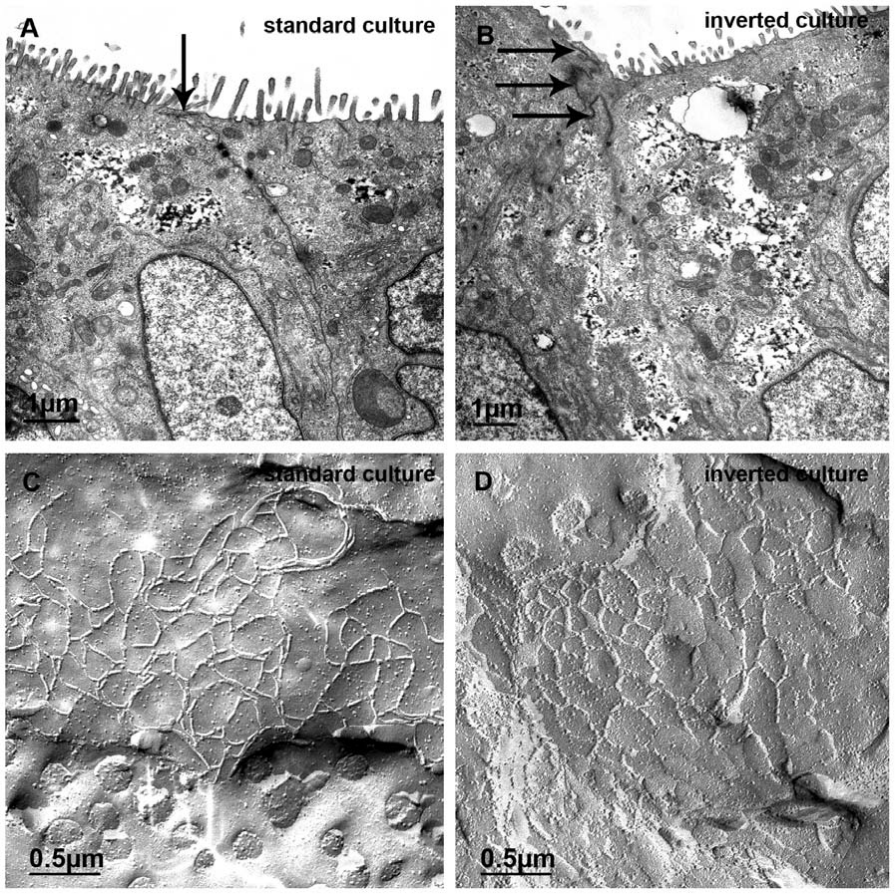
Electron microscopic analysis of HIBCPP TJ structure. HIBCPP were grown on transwell filter supports in the standard (**A**, **C**) and the inverted (**B**, **D**) culture system, respectively. Transmission electron microscopy studies (**A**, **B**) show that in both culture systems the cells are connected by TJs (arrows), which are located close to the apical side as indicated by the presence of microvilli. Examination of HIBCPP by freeze fracture electron microscopy (**C**, **D**) revealed a broad band of closely meshed TJ strands. The diameter of meshes were in the magnitude of 0.2 to 0.4 µm.

To obtain a more detailed impression of the morphology of the HIBCPP transwell filter cultures we analyzed samples with a TEER around 500Ω×cm^2^ by transmission as well as freeze fracture electron microscope (Fig. 5). Transmission electron microscopic images reveal the presence of TJs between the cells close to the apical border in both cell culture systems (Fig. 5A,B). Also, microvilli can be seen at the apical cell side. Further analysis of TJ structure by freeze fracture electron microscopy shows that the TJs form a broad band of meshed TJ strands, which did not differ between standard and inverted cultures (Fig. 5C,D).

### Polar invasion of *S. suis* into HIBCPP layers

**Figure 6:**
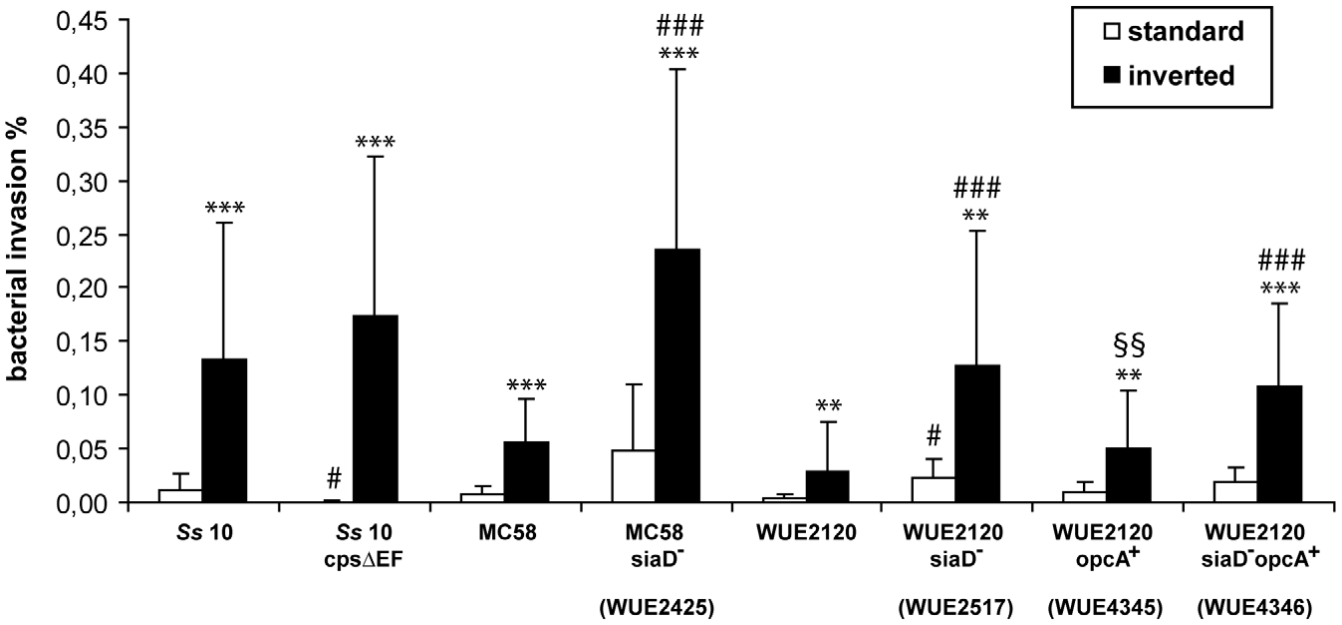
Polar invasion of bacteria into HIBCPP. Invasion of *S. suis* and *N. meningitidis* strains was analysed by double immunofluorescence. Standard (white bars) and inverted (black bars) transwell filter cultures were infected with the indicated strains for 4 h before immunofluorescence staining and quantification of invasion as described in Experimental procedures. Data shown are the mean+SD of a minimum of four independent experiments each performed at least in duplicates. ** (highly significant; p<0.01), *** (extremely significant; p<0.001); when invasion in the inverted filter system compared to invasion in the standard filter system. # (significant; p<0.05), ### (extremely significant; p<0.001); when invasion of the unencapsulated strain compared to invasion of the capsulated strain in the standard or inverted filter system, respectively. §§ (highly significant; p<0.01); when invasion of the Opc complemented strain compared to the respective not complemented strain.

Experimental studies employing the zoonotic pathogen *S. suis* as model organism had shown that *S. suis* can invade into and translocate through PCPEC [28]. We now investigated invasion of wild type *S. suis* strain 10 into HIBCPP grown on Transwell filters by double immunofluorescence microscopy as described in Experimental procedures. Quantitative analysis of invaded bacteria after 4 h treatment with an MOI of 10 revealed that *S. suis* strain 10 displays a significantly stronger invasion when applied in the inverted culture system compared to the standard culture, demonstrating polar invasion by *S. suis* into HIBCPP (Fig. 6). The TEER of all investigated transwell filter cultures stayed stable during the period of experimentation and assay conditions were non-cytotoxic as determined by Life/Dead assay (data not shown). The invasion level of S. *suis* strain 10 into HIBCPP was in the order of magnitude of that obtained in the porcine system.

To analyse the influence of the capsule of *S. suis* during invasion into HIBCPP we infected HIBCPP with the *S. suis* strain 10 cpsΔEF, an isogenic acapsular mutant of strain 10 [40]. As observed with the wild type, strain 10cpsΔEF invaded PCPEC in a polar fashion only from the basolateral side (Fig. 6). Although this invasion was not statistically different from that obtained with the wild type, statistical analysis indicated a trend (p = 0.0522) towards a stronger invasion by the acapsular mutant. Strain 10 did enter HIBCPP in the standard culture significantly better than the acapsular mutant (p = 0.0314), but invasion levels were very low with both strains.

**Figure 7:**
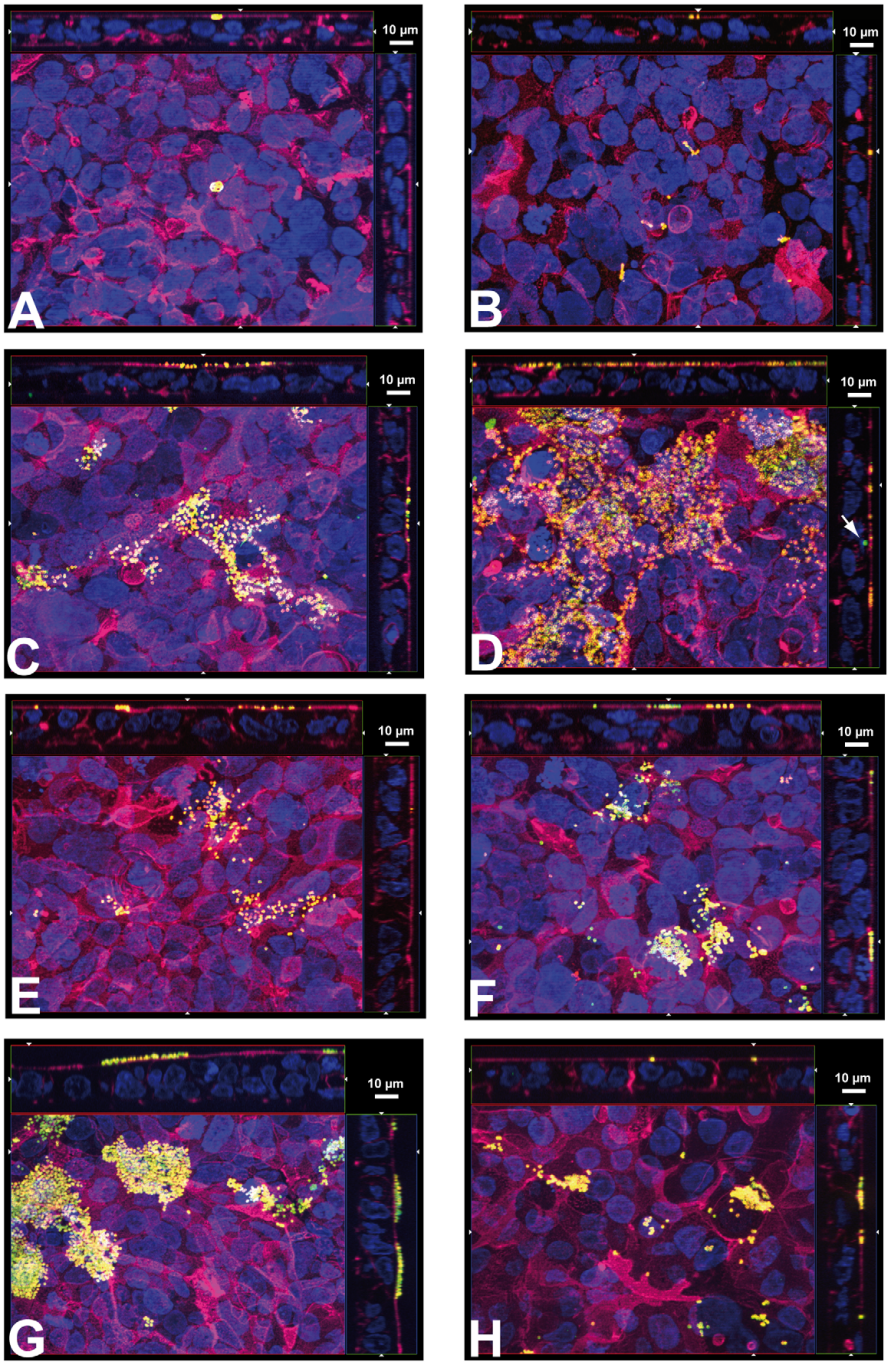
Double immunofluorescence microscopy of adherence and invasion of HIBCPP standard cultures infected with *S. suis* and *N. meningitidis*, respectively. HIBCPP grown in the standard culture system were inoculated with the indicated bacteria (MOI 10) and after 4 h subjected to double immunofluorescence microscopy to detect intracellular (green) and extracellular (yellow) bacteria. Cell nuclei were 4,6-diamidino-2-phenylindole (blue)-stained. The actin cytoskeleton was visualized with phalloidin (purple/magenta). Apotom images: bottom of each panel is an *xy* en face view of HIBCPP shown in a maximum-intensity projection through the *z*-axis of selected slices; top and side of each panel is a cross-section through the *z*-plane of multiple optical slices. The apical side of HIBCPP is oriented towards the top or the right side, respectively, of the top and side images of each panel. *S. suis* strains 10 (**A**) and 10 cpsΔEF (**B**) show adherence to the apical membrane but no invasion. *N. meningitidis* serogroup B strain MC58 (**C**) and its isogenic acapsular mutant (**D**) display strong adhesion, but only rare invasion events (arrows). Similarly, *N. meningitidis* serogroup C strain WUE2120 (**E**) and its derivatives WUE2517 (siaD^−^) (**F**), WUE4345 (opcA^+^) (**G**) and WUE4346 (siaD^−^ opcA^+^) (**H**) adhere strongly to the apical membrane; invasion is rarely detected. Shown are representative examples of four independent experiments, which gave similar results.

**Figure 8:**
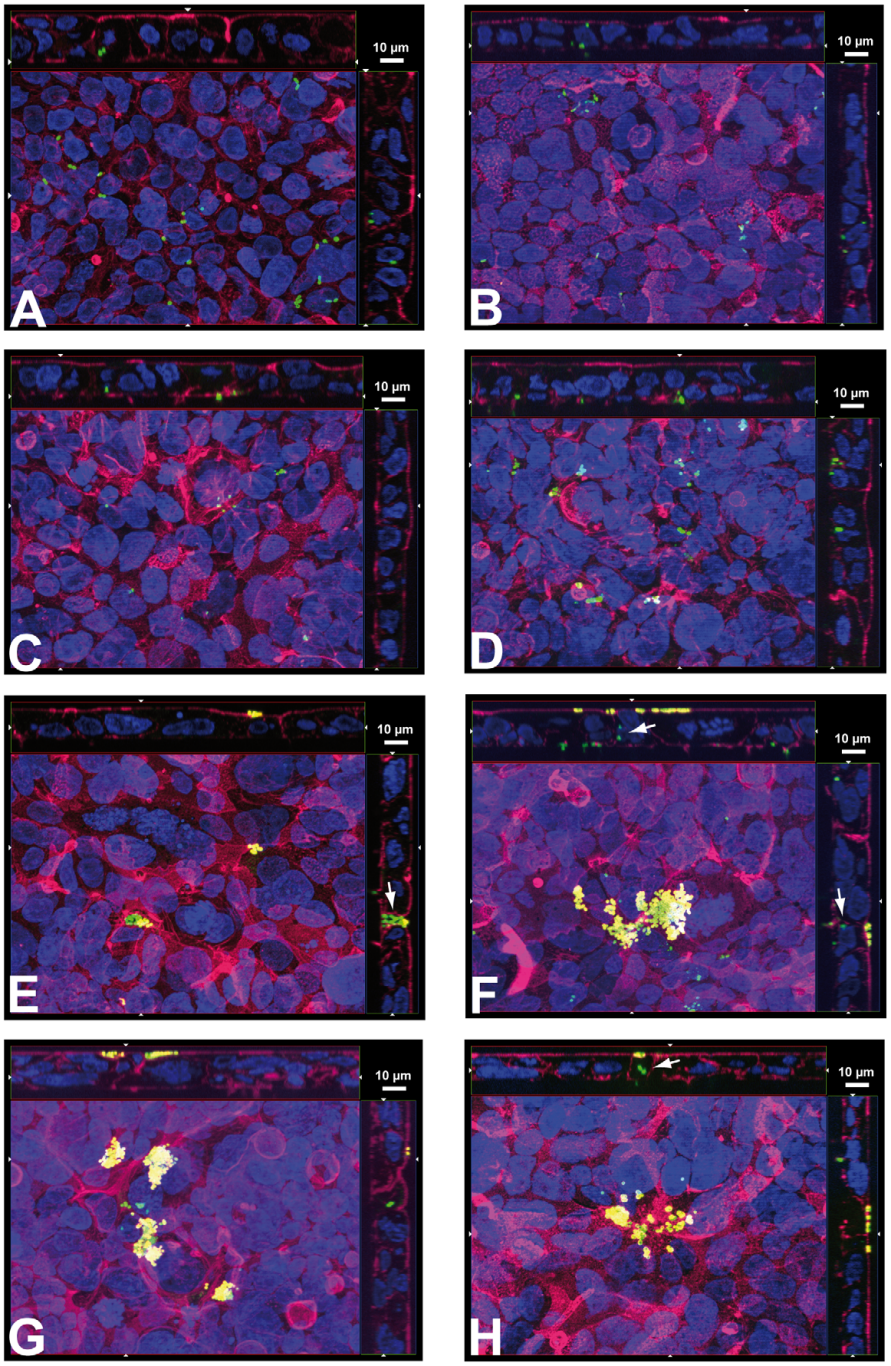
Double immunofluorescence microscopy of adherence and invasion of HIBCPP inverted cultures infected with *S. suis* and *N. meningitidis*, respectively. HIBCPP grown in the inverted culture system were inoculated with the indicated bacteria and analysed for intracellular (green) and extracellular (yellow) bacteria as described in Fig. 7. *S. suis* strains 10 (**A**) and 10 cpsΔEF (**B**) invade HIBCPP from the basolateral side. Similarly, basolateral invasion is observed for *N. meningitidis* serogoup B strain MC58 (**C**) and its isogenic acapsular mutant (**D**). *N. meningitidis* serogroup C strain WUE2120 (**E**) and its derivatives WUE2517 (siaD^−^) (**F**), WUE4345 (opcA^+^) (**G**) and WUE4346 (siaD^−^ opcA^+^) (**H**) invade HIBCPP from the basolateral side and form microcolonies at the apical membrane. Arrows indicate invaded bacteria directly below apically adhered microcolonies. Shown are representative examples of four independent experiments, which gave similar results.

Although *S. suis* strains 10 and 10 cpsΔEF invaded HIBCPP in only minimal numbers in the standard culture system bacteria adhered to the apical membrane of HIBCPP as demonstrated by analysis of the Apotome immunofluorescence images (Fig. 7A, B). In contrast, basolaterally invaded bacteria could easily be detected inside of HIBCPP in the inverted culture (Fig. 8A, B). Basolaterally adhered *S. suis* were rarely detected in this culture system (data not shown). It should be noted that in the inverted culture the analysis of adherence to the basolateral side is limited due to the presence of the filter membrane, which restricts the cell surface accessible for bacterial adhesion to the filter pores. Noteworthy, in the inverted culture system some adherent bacteria could be detected at the apical side of HIBCPP indicating translocation from the basolateral (blood) to the apical (CSF) side (data not shown).

### Invasion of *N. meningitidis* into HIBCPP is polar and attenuated by the presence of a capsule

The Gram-negative bacterium *N. meningitidis* is a major cause of meningitis world wide [23]. Importantly, the choroid plexus has been discussed as entry site for *N. meningitidis* into the brain [31], [32]. *N. meningitidis* encodes several adhesins, which are involved in interactions of the bacteria with host cells. Major adhesins include, besides the polymeric pili, the outer membrane opacity proteins Opa and Opc [41]. Since *N. meningitidis* is an obligate human bacterium HIBCPP provide an appropriate experimental setting to investigate the invasion properties at the BCSFB. For our studies we selected the well described serogroup B strain MC58 [42] as well as the pathogenic serogroup C strain WUE2120 [43]. Strain WUE2120 belongs to the meningococcal lineage of the ST-11/ET-37 complex, which does not possess an *opc* allele and therefore does not express Opc [44]. To analyse the role of Opc in more detail strain WUE2120 was complemented with the *opcA* gene from MC58 by transformation with an *opcA* expression plasmid. Finally, to decipher the role of the capsule in meningococcal context unencapsulated mutants of the *N. meningitidis* strains MC58 (WUE2425) and WUE2120 (WUE2517) were employed in our assays as well as strains complemented with Opc, i.e. WUE4345 (WUE2120 opcA^+^ (pHC47)) and WUE4346 (WUE2120 siaD^−^ opcA^+^ (pHC47)).

To determine invasion rates HIBCPP were infected with all *N. meningitidis* strains for 4 h using an MOI of 10 and analysed by double immunofluorescence assays as described in Experimental procedures. As with *S. suis* TEER values of investigated filter cultures stayed stable and assay conditions were non-cytotoxic (data not shown). Also, we generally observed invasion rates comparable to those obtained with *S. suis*. Quantitative analysis of invaded bacteria revealed for all investigated *N. meningitidis* strains significantly higher invasion rates when applied in the inverted culture system compared to the standard culture (Fig. 6), pointing to polar basolateral invasion by *N. meningitidis*. Complementation of strain WUE2120 and its acapsular mutant with Opc did lead to significantly higher invasion with the complemented wild type strain, but not the complemented mutant strain, from the basolateral side in the inverted culture system (p = 0.0086). Results obtained during control experiments performed with strains WUE2120 and WUE2517 transformed with the empty plasmid pAP1 did not give results different from those obtained with the untransformed strains (data not shown).

Examining the influence of the capsule during meningococcal invasion we observed significantly stronger invasion into HIBCPP from the basolateral side for all acapsular *N. meningitidis* strains compared to their encapsulated counterparts in the inverted transwell culture system (p<0.0001 for WUE2425 (MC58 siaD^−^); p<0.0001 for WUE2517 (WUE2120 siaD^−^); p = 0.0008 for WUE4346 (WUE2120 siaD^−^ opcA^+^ (pHC47)). Invasion from the apical side in the standard culture was significantly stronger only for acapsular strain WUE2517 compared to the wild type strain WUE2120 (p = 0.0226).

### *N. meningitidis* form microcolonies at the apical side of HIBCPP after basolateral invasion and translocation

It has been described that after attachment *N. meningitidis* forms microcolonies on the surface of target cells [23]. Visual analysis of Apotome immunofluorescence images taken from HIBCPP after infection with *N. meningitidis* revealed that all strains employed in our study adhered in large amounts to the apical surface of HIBCPP in the standard culture system (Fig.7C–H). The observed pattern of meningococcal attachment is in agreement with the formation of microcolonies. With all strains analysed invaded bacteria could rarely be detected (e.g. arrow in Fig. 7D, and data not shown), reflecting the scarce amount of invasion from the apical side of HIBCPP quantified in Fig. 6.

To analyse invasion of *N. meningitidis* from the basolateral side we similarly performed in-depth analyses of Apotome immunofluorescence images from HIBCPP grown in the inverted culture system after infection with *N. meningitidis*. As with *S. suis* basolaterally adhered bacteria were rarely detected in this system (data not shown). In contrast, we readily detected invaded bacteria with all *N. meningitidis* strains investigated (Fig. 8). Especially with strain WUE2120 and its derivatives we regularly observed bacterial microcolonies at the apical side of the basolaterally infected HIBCPP, i.e. the formation of microcolonies at the apical cell side occurs after *N. meningitidis* has fully invaded and transmigrated through the cells. Images presenting microcolonies are provided in Fig. 8C, D, E and F. In several cases we observed invaded bacteria directly below the apically attached microcolonies (e.g. arrows in Fig. 8C, D and F, and data not shown). In some cases apically adhered bacteria in the inverted culture system were also detected for strain MC58 and its unencapsulated mutant WUE2425 (data not shown).

**Figure 9:**
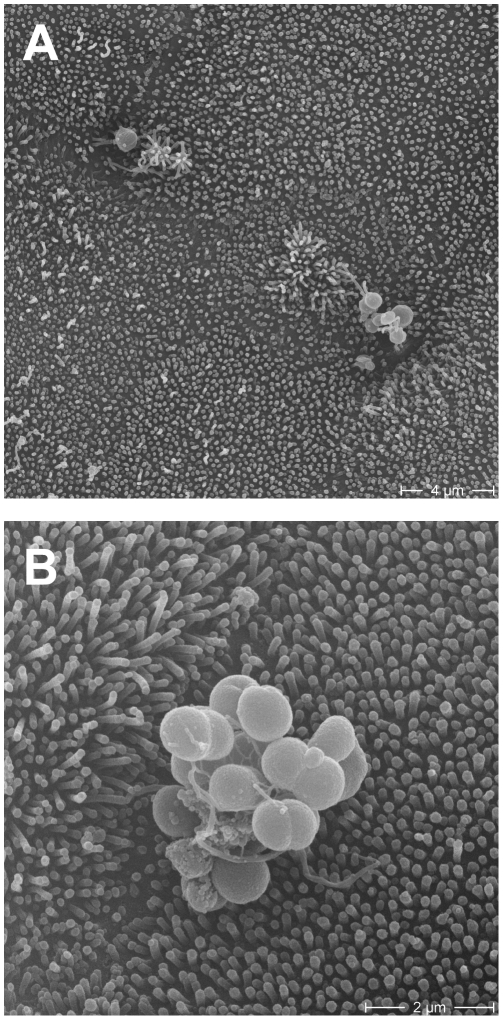
Analysis of transmigrated *N. meningitidis* attached to the apical membrane of HIBCPP. HIBCPP grown in the inverted system were infected with *N. meningitidis* strain WUE4346 (siaD^−^ opcA^+^) at an MOI of 100 (**A**) or 10 (**B**) for 4 h and subsequently analysed by scanning electron microscopy. Single bacteria (**A**) and microcolonies (**A**, **B**) are located at the apical membrane, which can be identified by the presence of microvilli.

We further confirmed the presence of microcolonies formed by transmigrated *N. meningitidis* by analysing bacterially infected HIBCPP cultivated in the inverted culture system by scanning electron microscopy. Fig. 9A shows a single bacterium and a small microcolony of *N. meningitidis* strain WUE4346 (WUE2120 siaD^−^ opcA^+^ (pHC47)) attached to the apical membrane, which can be clearly identified by the presence of microvilli. A larger microcolony is also shown in close-up in Fig. 9B, proving the presence of transmigrated bacteria at the apical side of HIBCPP.

## Discussion

The epithelial cells of the choroid plexus constitute the morphological correlate of the BCSFB [3] and experimental evidence points to the choroid plexus as an entry gate into the CSF for several bacterial pathogens including *N. meningitidis*, *E. coli* and the zoonotic pathogen *S. suis* [24]–[27], [31], [32]. Since the pathogenesis of bacterial meningitis is still poorly understood a suitable human model of the BCSFB would be highly supportive for progress in both basal and industrial research.

In this study we establish the recently characterized human choroid plexus papilloma cell line, HIBCPP, as BCSFB model. Morphologically, the HIBCPP have been described as an epithelial cell arrangement with a monolayer system. They exhibit pleomorphic and neoplastic features, lack contact inhibition and can overlap each other [22]. In the present manuscript, we show now that after implementing optimally adjusted culture conditions HIBCPP display fundamental properties of a functional BCSFB *in vitro*. Most importantly, HIBCPP present high TEER values when grown on transwell filter supports, which are in the order of magnitude of values observed *in vivo* in animal models [45], [46]. Additionally, HIBCPP develop a low permeability for the paracellular flux of macromolecules. The development of a sufficiently high TEER concomitantly with an impermeability for macromolecules requires the formation of continuous functional TJs, which is a crucial feature of a valid BCSFB model and is lacking by a described choroid plexus carcinoma cell line, the only other human choroid plexus cell line characterized as BCSFB model so far [19]–[21]. Consistent with the development of a barrier function, immunofluorescence as well as electron microscopic analyses clearly demonstrate the presence of continuous apical TJs in the HIBCPP *in vitro* system. A polar epithelial phenotype of HIBCPP is also confirmed by the presence of microvilli at the apical membrane. Further underlining their qualification as BCSFB model, HIBCPP express Transthyretin, IGF2 and FOXJ1, which have been described as choroid plexus epithelial cell markers [36]–[39].

Despite this cumulative evidence that HIBCPP present a relevant *in vitro* BCSFB model, its qualification for research purposes, e.g. infection studies, needed to be confirmed. Recently we had analysed the mechanisms of bacterial invasion and translocation at the BCSFB, employing *S. suis* and a primary porcine system [28]. In the present study we could confirm the important observation of polar invasion of *S. suis* only from the physiologically relevant basolateral side in the human system, pointing to consistency in fundamental properties of BCSFB models. Even more important, polar invasion into HIBCPP from the basolateral side occurred also after challenge with the obligate human bacterium *N. meningitidis*, an observation that points to a more general characteristic of choroid plexus epithelial cells regarding the entry of bacterial pathogens. *N. meningitidis* did not invade HIBCPP from the apical side, although all meningococcal strains displayed intense adhesion and formation of microcolonies at the apical side of HIBCPP in the standard culture system. The zoonotic agent *S. suis* showed, in contrast to the porcine barrier system [28], only low adherence at the apical side of HIBCPP.

Entry by *N. meningitidis* into HIBCPP from the basolateral side was consistently lower in the presence of capsule. Increase of invasion rates by capsule-deficient meningococci compared to the wild-type has also been shown for epithelial cell lines and human umbilical vein endothelial cells (HUVECS) [47], as well as for HBMEC [48]. Furthermore, enhanced invasion of the acapsular mutant was demonstrated for *S. suis* into PCPEC from the basolateral side [28]. Basolateral invasion of *S. suis* into HIBCPP in the inverted culture system showed a trend (p = 0.0522) towards higher invasion of the capsule-deficient strain. Apical invasion in the standard filter system was higher with the encapsulated wild type, but it should be noted that invasion levels were extremely low from the apical side with both strains. It is conceivable that capsule expression is required for survival in the blood, a notion that is supported by the observation that acapsular *S. suis* is avirulent in mice and pigs [40], [49], [50]. Capsule expression of *S. suis* or *N. meningitidis* could be down-regulated upon contact with host cells, a phenomenon that has been described for *N. meningitidis* and *Streptococcus pneumoniae* (*S. pneumoniae*) [51], [52], e.g. to support invasion.

Invasion of *N. meningitidis* into HBMEC has been shown to involve interactions of the Opc protein with endothelial integrins by interaction with the serum proteins vitronectin and fibronectin [41], [48], [53]. In contrast, adhesion and invasion of epithelial cells by *N. meningitidis* does not require the presence of serum factors but rather binding of Opc-expressing meningococci to heparin sulphate proteoglycan receptors [54], [55]. During our experiments performed in the absence of human serum factors we observed a significantly increased invasion of *N. meningitidis* strain WUE2120 complemented with the *opcA* gene of strain MC58, suggesting involvement of Opc during cellular entry, although Opc complementation of the acapsular mutant of strain WUE2120 did not lead to enhanced invasion. To decipher the exact influence and role of Opc during the invasion of HIBCPP, e.g. involvement of serum factors, further investigation is required.

One of the most intriguing findings of our study is the detection of bacteria as microcolonies at the apical surface of HIBCPP basolaterally infected with *N. meningitidis*, which demonstrates the transmigration of bacteria from the basolateral to the apical side. This observation is in strong contrast to the absence of microcolonies formed by *S. suis* in this experimental setup, although some apically adhered and therefore transmigrated *S. suis* could be detected, and points to different survival strategies of *N. meningitidis* and *S. suis* after entry into the CNS. Noteworthy, microcolony formation is a common step during the colonization and invasion of *N. meningitidis* at the human nasopharynx [23]. Initial contact of meningococci with nasopharyngeal epithelial cells is mediated by type IV pili, the receptor for which may be the I-domain of integrin alpha chains or possibly CD46 [56]. Meningococci proceed to proliferate on the surface of human nonciliated epithelial cells, forming small microcolonies at the site of initial attachment. Due to reorganization of the host cell surface meningococcal microcolonies are remarkably resistant to mechanical stress [57]. To our knowledge our study is the first demonstration that meningococci or other bacteria have the capability to form microcolonies after translocation through a host cell layer. Microcolonies represent bacterial proliferation and consecutive aggregation at the apical surface, and microcolony formation might protect the bacterial community from immune factors of the host. Still, the exact relevance of microcolony formation at the apical membrane of choroid plexus epithelial cells is not clear and might be just a consequence of the normal growth behaviour of *N. meningitidis*.

In summary, employing the HIBCPP cell line we establish the first functional human BCSFB model. Using transwell filter cultures we demonstrate polar bacterial invasion only from the basolateral side, confirming results obtained in a primary porcine system. We show that basolateral invasion of *N. meningitidis* is capsule regulated, and invaded *N. meningitidis* forms microcolonies at the apical membrane, providing novel insight into the mechanism of meningococcal translocation at the BCSFB during meningitis. The HIBCPP model holds promise for research in a wide range of applications in basic as well as industrial research. These applications include, but are not limited to, research in infectious diseases and pharmacology.

## Experimental Procedures

### Bacterial strains and growth conditions

Bacterial strains used in this study are listed in Table 1. *S. suis* Serotype 2 virulent strain 10 and its isogenic non-encapsulated mutant strain 10cpsΔEF [40] were kindly provided by H. Smith (DLO-Institute for Animal Science and Health, Lelystad, the Netherlands). *S. suis* strains were maintained as stock cultures in Todd-Hewitt broth (THB; Oxoid, Wesel, Germany) containing 10% glycerol at −80°C. For the experiments 100 µl of the bacteria stocks were inoculated in 10 ml THB and incubated at 37°C for about 6 h with mild agitation in order to reach the mid-log phase. *S. suis* cultures were washed twice with HIBCPP-medium (DMEM/HAM's F12 1∶1 supplemented with 4 mM L-Glutamine and 5 µg ml^−1^ insulin) without antibiotics (Ab) and were adjusted to an optical density at 600 nm (OD_600_) of 0.65. This stock solution contained approximately 2×10^8^ colony-forming units (CFU) ml^−1^ and was used for the assays at appropriate dilutions.

**Table 1:** Bacterial strains used in this study.

Bacterial strain		Synonym	Serotype/serogroup	Characteristic(s)	Source or reference
	10		2	wild-type	[40]
	10cpsΔEF		unencapsulated	isogenic cpsEF mutant of strain 10	[40]
*N. meningitidis*	MC58		B	wild-type	[42]
	MC58 siaD^−^	WUE2425	unencapsulated	isogenic *siaD* mutant of MC58	[61]
	WUE2120		C	wild-type	[43]
	WUE2120 siaD^−^	WUE2517	unencapsulated	isogenic *siaD* mutant of WUE2120	[61]
	WUE2120 opcA^+^ (pHC47)	WUE4345	C*S. suis*	complemented with *opcA* from MC58	this study
	WUE2120 siaD^−^ opcA^+^ (pHC47)	WUE4346	unencapsulated	isogenic *siaD* mutant of WUE2120 complemented with *opcA* from MC58	this study
	WUE2120 pAP1	WUE4624	C	transformed with plasmid pAP1	this study
	WUE2120 siaD^−^ pAP1	WUE4625	unencapsulated	isogenic *siaD* mutant of WUE2120 transformed with plasmid pAP1	this study

*N. meningitidis* serogroup C strains WUE4345 (WUE2120 opcA^+^ (pHC47)) and WUE4346 (WUE2120 siaD^−^ opcA^+^ (pHC47)) were generated by transformation of the plasmid pHC47 expressing the *opcA* gene from *N. meningitidis* MC58 inserted into the plasmid pAP1 [58] and selected by addition of erythromycin (7 µg ml^−1^). The Expression of Opc in the complemented strains was verified by Western blotting after generation of the strains. The Opc expression plasmid pHC41 is stable in the complemented strains. Additionally, for analysis the Opc complemented strains were grown in the presence of selective antibiotic until start of the experiment. As control strains WUE2120 and and its unencapsulated mutant WUE2517 (WUE2120 siaD^−^) were transformed with the empty plasmid pAP1 thereby generating strains WUE4624 (WUE2120 pAP1) and WUE4625 (WUE2120 siaD^−^ pAP1), respectively. All used *N. meningitidis* strains were maintained as stock cultures in HIBCPP-medium without Ab containing 10% glycerol at −80°C. For experiments, bacteria were cultivated overnight at 37°C and 5% CO_2_ on chocolate agar plates supplemented with 1% Polyvitox (Oxoid, Wesel, Germany). Bacteria were washed twice with HIBCPP-medium without Ab and adjusted to an optical density at 600 nm (OD_600_) of 1.00. This stock solution contained approximately 1×10^9^ CFU/ml and was further diluted in fresh HIBCPP-medium without Ab containing 1% FCS for the experiments.

### Cultivation of HIBCPP on transwell filter

HIBCPP were cultured in DMEM/HAM's F12 1∶1 supplemented with 4 mM L-Glutamine, 5 µg ml^−1^ insulin, penicillin (100 U ml^−1^) and streptomycin (100 µg ml^−1^), 15% heat inactivated fetal calf serum (FCS) [HIBCPP-medium with 15% FCS]. Since HIBCPP have been described to change doubling time with increasing passages [22] only cells between passage 33 and 37 were used. For filter-based assays the amounts of cells indicated in the respective experiments were seeded on transwell filters (pore diameter 3.0 µm, pore density 2.0×10^6^ pores per cm^2^, membrane diameter 0.33 cm^2^; Greiner Bio-One, Frickenhausen, Germany). For the standard transwell filter system cells were seeded into the upper filter well. Since HIBCPP can form papillary-like structures and grow in multilayers [22], trypsinization and seeding of HIBCPP were extensively optimized to allow formation of a maximal proportion of a monolayer on the transwell filters. Subsequently, cells were washed once each of the following two days. Medium was added to the lower well not before day two after seeding. For the inverted transwell filter system the cells were basically treated as described previously for PCPEC [28]. In detail, the cells were seeded on transwell filters that were flipped over and placed in a medium-flooded 12-well plate. Cells were fed the following day and the filters were flipped over again and placed in a 24-well plate on day 2 after seeding.

Upon confluence, HIBCPP had a seeding density of approximately 1.21×10^6^ cells cm^−2^ (evaluated by 4,6-diamidino-2-phenylindole staining of the cell nuclei using immunofluorescence imaging). When TEER values became greater then 70Ω×cm^2^, cell culture was continued in HIBCPP-medium containing 15%, 1% or 0% FCS as indicated in the respective experiments. Cells were used for experiments 1 or 2 days later when the TEER was around 500Ω×cm^2^.

### Infection of HIBCPP with bacteria and measurement of TEER

The TEER of HIBCPP grown on Transwell filters was measured using an epithelial tissue voltohmmeter using the STX-2 electrode system (Millipore, Schwalbach, Germany). For infection studies HIBCPP were cultivated on Transwell filters until TEER values became greater then 70Ω×cm^2^. Subsequently, cell culture was continued in HIBCPP-medium without Ab containing 1% FCS. HIBCPP with a TEER around 500Ω×cm^2^ were exposed to bacteria with a multiplicity of infection (MOI) of 10 (except where indicated otherwise) in HIBCPP-medium without Ab containing 1% FCS from the upper compartment of the transwell filter, which allows infection of HIBCPP from the apical side in the standard transwell filter system and from the basolateral side in the inverted transwell filter system. The MOI of the bacteria were calculated based on the number of cells per well at confluence (1.21×10^6^ cells cm^−2^). Following infection, TEER across the cell layers was monitored over a range of 4 h. Resistance values of cells on Transwell filters in medium alone were used as control values.

### Determination of paracellular permeability

Paracellular permeability of HIBCPP monolayer was essentially determined as described previously [28]. Briefly, the passage of a FITC-inulin (Sigma, Deisenhofen, Germany) tracer solution (100 µg ml^−1^, average molecular weight, 3000–6000) from the apical to the basolateral compartment of transwell filters was measured in a Tecan Infinite M200 Multiwell reader (Tecan, Switzerland). When cells were infected with bacteria the FITC-inulin flux was determined over a range of up to 4 h post-infection.

### Immunohistochemistry

Immunohistochemistry was basically performed as described previously for PCPEC [33]. HIBCPP were plated and observed in transwell filters. For phalloidin staining of the actin cytoskeleton as well as tight junction staining of ZO-1 and Occludin HIBCPP were fixed with 4% formaldehyde (w/v in PBS) at room temperature for 10 min, whereas for Claudin-1 staining HIBCPP were fixed with ice-cold methanol for 20 min. Subsequently, HIBCPP were permeabilized by applying 0.5% Triton X-100/1% BSA (v/v in PBS). Immunofluorescence staining was performed using primary antibodies (polyclonal rabbit anti-ZO-1, anti-Occludin, or anti-Claudin-1 at 1∶250 dilution over night at 4°C) obtained from Zymed (San Francisco, USA) and fluorophor-labelled secondary antibodies (polyclonal chicken anti-rabbit-IgG Alexa Fluor 594 at 1∶250 dilution for 1 h at 4°C) obtained from Molecular Probes (Oregon, USA). Actin was stained by incubating the cells with phalloidin Alexa Fluor 488 (1 U 300 µl^−1^; Molecular Probes, Oregon, USA) for 60 min at 4°C. Nuclei were stained with 4′-6-diamidino-2-phenylindole dihydrochloride (DAPI) (1∶50000). Images were acquired with Zeiss Apotome and Axiovision software (Carl Zeiss, Jena, Germany) using a 63×/1.4 objective lens. This system provides an optical slice view reconstructed from fluorescent samples. For graphical presentation representative selections of images were chosen from multiple standard microscopic fields. All immunofluorescence experiments were performed on transwell filters at least in duplicate for each value and repeated at least three times.

### Determination of bacterial invasion by double immunofluorescence

This was done as previously described elsewhere [59] with some modifications. HIBCPP were grown on transwell filters and infected with *N. meningitidis* or *S. suis* as described above. After 4 h infected cells were washed three times with HIBCPP medium without Ab containing 1% BSA (bovine serum albumin) (blocking buffer) and blocked with the same buffer for 20 min at 4°C to block non-specific binding sites. Blocking buffer was removed and preparations were incubated with the following primary antibodies for 20 min at 4°C at a dilution of 1∶200 (rabbit-anti-*N. meningitidis* α-OMP) or 1∶100 (all other primary antibodies): *S. suis* strain 10 and strain 10cpsΔEF, goat-anti-*S. suis* [28]; *N. meningitidis* MC58 and MC58 siaDmut, rabbit-anti-*N. meningitidis* α-OMP; *N. meningit*i*dis* 2120 and derivatives, antibody against *N. meningitidis* generated in chicken (chicken-anti-*N. meningitidis*). Then cells were washed again with HIBCPP medium without Ab containing 1% BSA and hereafter fixed for 10 min with 4% formaldehyde and washed with PBS. Subsequently, the formaldehyde-fixed transwell filter membrane preparations were cut out of the insert and were washed with PBS/1% BSA. After washing with blocking buffer, samples were incubated for 15 min with either Alexa Fluor 594 (red) donkey anti-goat, chicken anti-rabbit or goat-anti-chicken antibodies (each 1∶500; Molecular Probes, Oregon, USA) when *S. suis or N. meningitidis* were analyzed, respectively, to stain extracellular bacteria. In the following, the preparations were washed with blocking buffer and epithelial cells were permeabilized with PBS/0.5% Triton X-100/1% BSA for 60 min at room temperature and washed with blocking buffer again. The preparations were then incubated with the following primary antibodies for 30 min at room temperature at a dilution of 1∶200 (rabbit-anti-*N. meningitidis* α-OMP) or 1∶100 (all other primary antibodies): *S. suis* strain 10 and strain 10cpsΔEF, rabbit anti-*S. suis* [28]; *N. meningitidis* MC58 and MC58 siaDmut, again rabbit-anti-*N. meningitidis* α-OMP; *N. meningitidis* 2120 and derivatives, again chicken-anti-*N. meningitidis*. Samples were subsequently washed with blocking buffer, and incubated with either Alexa Fluor 488 (green) chicken anti-rabbit or goat-anti-chicken antibodies (1∶500; Molecular Probes, Oregon, USA) for 20 min to stain intra- and extracellular bacteria. Cells were washed again and stained with Phalloidin Alexa Fluor 660 (Molecular Probes, Oregon, USA) and 4′-6-diamidino-2-phenylindole dihydrochloride (DAPI) (1∶50000) to stain cell nuclei for 1 h at room temperature. After final washing, the filter membranes were embedded in ProLongAntifadeReagent (Invitrogen, Karlsruhe, Germany), and stored at 4°C until examination. All antibodies were diluted in blocking buffer. Each washing step was repeated three times. Images were acquired with Zeiss Apotome and Axiovision software (Carl Zeiss, Jena, Germany) using a 63×/1.4 objective lens. The image acquisition was carried out using the Zeiss scanning software Axiovison 4.6 and Axiovison module Inside 4D. Invaded bacteria were determined per pre-defined field by counting 20 fields per filter membrane. The percentage of invaded bacteria was calculated as described in the following: the mean bacterial count of the 20 microscopic fields was multiplied with an area coefficient. The result, expressing the total amount of bacteria in 0.33 cm^2^ transwell filter, was divided by the amount of total bacteria grown in media during 2 h and 4 h and expressed as percentage invasion. Assays were performed at least in duplicates for each value and repeated at least four times.

### Reverse-transcriptase polymerase chain reaction

Total cellular RNA was isolated using the RNeasy mini kit (Qiagen, Hilden, Germany) and subsequently treated with RNase-free DNase I (Roche, Grenzach-Wyhlen, Germany). After spectrophotometrical determination of the RNA concentration 1 µg of total RNA was reverse transcribed with the Affinity Script QPCR cDNA synthesis kit according to the instructions provided by the manufacturer (Stratagene, La Jolla, CA). The following PCR reactions were performed with the Tag PCR core kit (Qiagen, Hilden, Germany) applying 0.5 µl of the generated cDNA again following the instructions provided by the manufacturer. PCR reaction mixtures were heated to 94°C for 2 min and then were subjected to 35 cycles of denaturation (94°C, 30 sec), annealing (60°C, 30 sec) and extension (72°C, 2 min) followed by a final extension step at 72°C for 7 min. Subsequently, PCR products were visualized by agarose gel electrophoresis and ethidium bromide staining. Primers employed for PCR amplification were 5′-GCCAAGCAATGGCAGTCTC-3′ and 5′-CTGGGCCGAAGAAATCCCATC-3′ for ZO-1, 5′-AACACCATTATCCGGGACTTCT-3′ and 5′-CGCGGAGTAGACGACCTTG-3′ for Claudin-3, 5′-ATCCAAGTGTCCTCTGATGGT-3′ and 5′-GCCAAGTGCCTTCCAGTAAGA-3′ for Transthyretin, 5′-CCTCCAGTTCGTCTGTGGG-3′ and 5′-CACGTCCCTCTCGGACTTG-3′ for insulin-like growth factor 2, 5′-CCTCCCTACTCGTATGCCAC-3′ and 5′-CGAGGCACTTTGATGAAGCAC-3′ for forkhead box J1, and 5′-GTTCGACAGTCAGCCGCATC-3′ and 5′-GGAATTTGCCATGGGTGGA-3′ for the house keeping gene glyceraldehyde-3-phosphate dehydrogenase (GAPDH). Some primers were generated using the PrimerBank resource [60]. Further primers for PCR amplification were 5′-AGGAACACATTTATGATGAGCAG-3′ and 5′-GAAGTCATCCACAGGCGAA-3′ for Occludin, 5′-GAAGATGAGGATGGCTGTCA-3′ and 5′-AAATTCGTACCTGGCATTGA-3′ for Claudin-1, 5′-CCTGCCAATCCCGATGA-3′ and 5′-TGCCCCATTCGTTCAAGTA-3′ for E-cadherin, and 5′-ACCATTCCTTGACGGTGTCTA-3′ and 5′-GCTGATTTTCCATTACGCCT-3′ for Claudin-2 and have been described before [21].

### Electron microscopy

For transmission electron microscopic analyses HIBCPP were grown on transwell filter employing either the standard or the inverted filter system. Filter with a TEER above 70Ω×cm^2^ were further cultivated in HIBCPP-medium 1% FCS and were used 1 or 2 days later. Filter with a TEER around 500Ω×cm^2^ were washed once in HIBCPP-medium with 0% FCS, fixed for at least 4 h in a 2% glutaraldehyd solution in 75 mM cacodylate buffer (pH 7.4) and again washed two times with 75 mM cacodylate buffer (pH 7.4). Subsequently, the support films were removed from the wells using a sharp ophthalmic scalpel. The filters were then cut into stripes and postfixed in 1% osmium tetroxide (OsO_4_) in cacodylate buffer for 1 h and dehydrated in ascending series of ethanol and propyleneoxide. For contrast enhancement, they were bloc-stained in uranyl-acetate in 70% ethanol for 4 h and flat-embedded in Araldite (Serva, Heidelberg, Germany). Using an ultramicrotome (Ultracut R, Leica, Bensheim, Germany), semi-(1 µm) and ultrathin sections (50 nm) were cut. Ultrathin sections were stained with lead citrate, mounted on copper grids and finally analysed with a Zeiss EM 10 (Oberkochen, Germany) electron microscope.

For freeze-fracturing, the filter stripes were treated with 30% glycerol and quick-frozen in nitrogen-slush (−210°C). Subsequently, the specimens were fractured in a Balzer's freeze-fracture device (BAF400D; Balzers, Liechtenstein) at 5×10^−6^ mbar and −150°C. Both complementary fracture faces were shadowed with platinum/carbon (2 nm, 45°) for contrast and carbon (20 nm, 90°) for stabilization of the replica. After removing the cell material in 12% sodium hypochlorite, the replicas were cleaned several times in double-distilled water and mounted on Pioloform-coated copper grids. The replicas were observed using an EM10A electron microscope (Carl Zeiss, Oberkochen, Germany). The pictures of ultrathin sections and freeze-fracture replicas were scanned at 300 dpi and processed with Adobe Photoshop.

For scanning electron microscopy samples were fixed with 2.5% glutaraldehyde in cacodylate buffer, postfixed with 1% osmium tetroxide (OsO_4_) in phosphate-buffered saline, dehydrated in a graded series of ethanol and critical-point-dried using CO_2_. Finally, the samples were sputter-coated with a layer of 7 nm gold/palladium (Bal-Tec MED 010) and examined at 20 kV accelerating voltage in a Hitachi S-800 field emission scanning electron microscope.

### Measurement of cell viability

Viality of the cells was measured using a Life/Dead assay (Invitrogen, Karlsruhe, Germany) according to the manufacturer's instructions. The results were photodocumented by fluorescence microscopy.

### Statistical analysis

For graphical presentations and statistical calculations, quantitative parameters are given by mean values and standard deviations. A 2-way ANOVA has been performed in order to test the influence of day and filter system on quantitative outcomes (TEER) simultaneously. Dunnett's test has been used to compare a medium containing 0% FCS or 1% FCS with a medium containing 15% FCS. Differences between two groups regarding the bacterial invasion (given in %) were tested by Mann Whitney U tests. A test result with p<0.05 has been considered as statistically significant. All statistical calculations have been performed with the SAS system, release 9.2 (SAS Institute Inc., Cary, NC, USA).
